# Reversible cerebral vasoconstriction syndrome: A narrative review

**DOI:** 10.1111/head.70048

**Published:** 2026-03-28

**Authors:** Ícaro Araújo de Sousa, Abner da Silva Machado, Arthur de Oliveira Veras, Thiago Oscar Goulart, Sarah Galassi Tatsuta, Trajano Aguiar Pires Gonçalves, Eva Rocha, Octávio Marques Pontes‐Neto

**Affiliations:** ^1^ Department of Neuroscience and Behavior Sciences, Medical School of Ribeirão Preto University of São Paulo Ribeirão Preto São Paulo Brazil; ^2^ Department of Biostatistics Harvard T.H. Chan School of Public Health Boston Massachusetts USA; ^3^ Department of Psychology, Faculty of Philosophy, Sciences and Letters Medical School of Ribeirão Preto Ribeirão Preto São Paulo Brazil; ^4^ Depatment of Neurology and Neurosurgery Federal University of São Paulo São Paulo São Paulo Brazil

**Keywords:** cognitive impairment, intracranial hemorrhage, stroke, thunderclap headache, vasoconstriction

## Abstract

**Objectives/Background:**

This review summarizes current insights into Reversible cerebral vasoconstriction syndrome (RCVS) diagnosis, management, and outcomes. RCVS is a cerebrovascular disorder characterized by recurrent thunderclap headaches and transient segmental vasoconstriction of cerebral arteries, typically resolving within 3 months.

**Methods:**

A comprehensive database search was performed across MEDLINE (via PubMed), Embase, Scopus, and the Cochrane Library.

**Results:**

Although often self‐limiting, RCVS may cause complications such as subarachnoid hemorrhage, ischemic stroke, and cerebral edema. Triggers include vasoactive substances, pregnancy, postpartum state, and physical or emotional stress. Differentiating RCVS from conditions like primary angiitis of the central nervous system, aneurysmal subarachnoid hemorrhage, and cerebral venous thrombosis is essential because clinical and imaging features may overlap, whereas treatments differ. Advances in neuroimaging, especially magnetic resonance angiography and vessel wall imaging, have enhanced diagnostic accuracy. Management focuses on eliminating triggers and symptomatic support. Calcium channel blockers are frequently used, although their impact on disease evolution remains uncertain.

**Conclusion:**

Although most patients recover without major sequelae, chronic symptoms such as long‐term headaches and neuropsychological symptoms, including cognitive impairment, underscore the need for ongoing follow‐up and suggest a post‐RCVS syndrome.

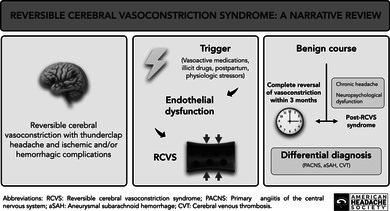

## Plain Language Summary

Reversible cerebral vasoconstriction syndrome (RCVS) causes sudden, severe “thunderclap” headaches due to temporary narrowing of brain arteries that typically resolves within 3 months, although serious complications like stroke can occur. This review examines the historical understanding of RCVS, its common triggers (including stress, physical exertion, certain medications, and pregnancy), and current approaches to diagnosis using advanced brain imaging techniques. A sudden, explosive headache requires immediate medical attention, and even after recovery, some patients experience ongoing headaches or cognitive changes that may indicate a “post‐RCVS” syndrome.

## Graphical abstract

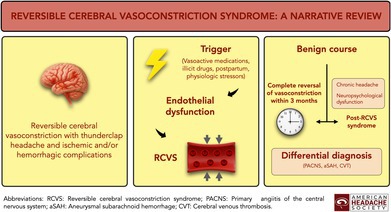

**Abbreviations**
3D‐RA: three‐dimensional rotational angiography
A1: first segment of anterior cerebral artery
A2: second segment of anterior cerebral artery
aSAH: aneurysmal subarachnoid hemorrhage
ASL: arterial spin labeling
BBB: blood‐brain barrier
CBF: cerebral blood flow
CGRP: calcitonin gene‐related peptide
cSAH: convexity subarachnoid hemorrhage
CSF: cerebrospinal fluid
CTA: computed tomography angiography
CVT: cerebral venous thrombosis
DSA: digital subtraction angiography
FLAIR: fluid‐attenuated inversion recovery
HELLP: hemolysis, elevated liver enzymes, and low platelet count
M1: first segment of middle cerebral artery
M2: second segment  of middle cerebral artery
MRA: magnetic resonance angiography
mRS: modified Rankin Scale
P2: second segment of posterior cerebral artery
PACNS: primary angiitis of the central nervous system
PIH: pregnancy‐induced hypertension
PRES: posterior reversible encephalopathy syndrome
RCVS: reversible cerebral vasoconstriction syndrome
REVERCE: reversible cerebral vasoconstriction syndrome international collaborative network
RNF213: ring finger protein 213
TCHs: thunderclap headaches
TGA: transient global amnesia
TKC: takotsubo cardiomyopathy
Val66Met: valine‐66‐methionine substitution in the BDNF gene
VWI: vessel wall imaging
WMHs: white matter hyperintensities

## INTRODUCTION

Reversible cerebral vasoconstriction syndrome (RCVS) is a condition characterized by recurrent thunderclap headaches (TCHs) and multifocal segmental narrowing of the cerebral arteries that resolves within 3 months. Although the clinical outcome is usually benign, neurological complications like convexity subarachnoid hemorrhage (cSAH), intraparenchymal hemorrhage, ischemic strokes, and edema can occur.^1^

**FIGURE 1:**
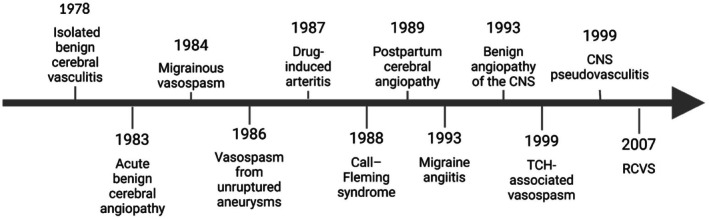
Terminologies in historical perspective: Over the past 5 decades, various terms have been used to describe entities marked by severe headaches, typically *thunderclap headaches* (TCHs) and *reversible cerebral vasoconstriction*, often triggered by medications or physiological and pathological conditions. Due to overlapping clinical features, these cases were historically misdiagnosed as PACNS, leading to unnecessary invasive interventions. In 2002, the syndromes were recognized as a unified group and could be linked to serotonergic triggers; and in 2007, the term *reversible cerebral vasoconstriction syndrome* (RCVS) was introduced, promoting “unity in diversity” by consolidating these previously distinct entities under a single, comprehensive classification.^1^, ^2^, ^3^ PACNS, primary angiitis of the central nervous system; RCVS, reversible cerebral vasoconstriction syndrome; TCHs, thunderclap headaches.

Since its first description in the 1960s, and the recognition in the 1970s of trigger‐related cases labeled “isolated benign cerebral vasculitis,” numerous other terms have been used to describe this condition, including *migrainous vasospasm*, *migraine‐angiitis*, *Call–Fleming syndrome*, *drug‐induced arteritis*, among others (Figure 1),^4^, ^5^, ^6^, ^7^, ^8^, ^9^, ^10^, ^11^, ^12^, ^13^, ^14^, ^15^, ^16^, ^17^, ^18^ reflecting the diverse clinical contexts in which RCVS can manifest. It was not until 2007 that Calabrese et al. proposed the term *reversible cerebral vasoconstriction syndrome* as an umbrella designation, establishing diagnostic criteria to unify these previously described entities.^2^

In recent years, there has been a significant increase in publications on RCVS, reflecting its growing recognition among neurovascular and headache specialists, although it is still an underdiagnosed disease entity.^19^ This surge has been driven by improved clinical characterization and advancements in neuroimaging, enabling more accurate diagnoses, and improving our understanding of its pathophysiology, epidemiology, triggers, and treatment options.^20^ Moreover, growing attention has been directed toward overlapping conditions associated with RCVS, such as posterior reversible encephalopathy syndrome (PRES) and transient global amnesia (TGA).^21^, ^22^ Additionally, emerging research has highlighted preliminary findings on various clinical features, including neuropsychological aspects.^23^

In this review, we explore the clinical and radiological features of RCVS, highlighting new clinical insights and offering a comprehensive overview of its diagnostic approach, differential diagnosis, and management.

## EPIDEMIOLOGY

RCVS has been reported across all continents, with large case series highlighting its wide spectrum of clinical manifestations and racial diversity. The true incidence of RCVS remains unknown; however, retrospective population‐wide studies estimate it at three cases per million per year.^24^ National Inpatient Sample data indicate that RCVS cases represent around 0.2 per 100,000 hospitalizations in the United States, with about 75% occurring in women.^25^ For instance, a large case series reported 67 patients at a single center over 3 years. This finding suggests that the condition is likely underestimated due to limited awareness and inconsistencies in medical coding.^1^ To address this issue, in 2016, RCVS was added to the International Classification of Diseases under code I67.841, a step expected to promote greater awareness, enable more comprehensive epidemiological studies, and help establish the true prevalence of the syndrome.^26^

Cases range from 4 months to 76 years, with a mean onset around 42 years.^27^, ^28^, ^29^, ^30^ Women tend to be older at presentation, have more frequent triggers, and show more severe manifestations than men.^31^ Pregnancy‐related RCVS accounts for roughly 8%–12% of cases, and affected patients are significantly younger than nonpregnant patients.^31^, ^32^ In children, RCVS is likely underrecognized; available series show a predominance in adolescents (7–16 years), about 80% male, and approximately one‐third with hematologic or rheumatologic disorders.^33^, ^34^

Ultimately, emerging exploratory data from the Reversible Cerebral Vasoconstriction Syndrome International Collaborative Network (REVERCE) suggest regional differences in which European cohorts show higher rates of significant brain lesions, unfavorable outcomes, secondary forms of disease, and absence of TCH. Although these findings may reflect selection bias related to differences in healthcare systems, further research is warranted to clarify the environmental, social, and genetic factors involved.^35^

Regarding the proportion of RCVS among intracranial arteriopathies, available data suggest a meaningful fraction. In a cohort of patients with angiographic abnormalities, 27% (30/110) received a final diagnosis of RCVS when a discriminative score was applied.^36^ In another series, digital subtraction angiography (DSA) with intra‐arterial vasodilator testing identified RCVS in nine of 26 patients (34.6%).^37^ These studies were not designed to estimate prevalence and have important limitations, including single‐center settings; complex referral populations; age restrictions; and small, likely selected, samples in which milder cases would not typically undergo DSA.^36^, ^37^, ^38^ Larger, multicenter studies with broader and more representative samples are needed to define this proportion more precisely.

## PUTATIVE TRIGGERS AND ASSOCIATED CONDITIONS

RCVS has been linked to a wide range of conditions, with the list of putative triggers expanding over time. However, the broad variability in nosology associated with RCVS suggests potential reporting bias, and patients are often exposed to multiple triggers or conditions simultaneously.^19^, ^39^ As a result, establishing a clear causal relationship remains challenging in many cases. Table 1 provides an overview of the reported triggers and associated conditions.

**TABLE 1:** Triggers and associated conditions.

Pregnancy and postpartum	Early puerperium, late pregnancy, eclampsia, pre‐eclampsia, HELLP syndrome, delayed postpartum eclampsia
Exposure to illicit drugs	Cannabis/marijuana, cocaine, ecstasy, amphetamine derivatives, LSD, nicotine patches, electronic cigarette, binge alcohol drinking
Headache disorders	Migraine, primary thunderclap headache, benign exertional headache, benign sexual headache, primary cough headache
Environmental exposure and physiologic conditions	High altitude, winter climate, cold water exposure, typhoons, coughing, sneezing, laughing, singing, swimming, severe grief, airplane descent
Medications, drugs, and toxins	Serotoninergic/antidepressants: SSRIs, SNRIs, triptans, bupropiom Sympathomimetic: phenylpropanolamine, pseudoephedrine, phenylephrine, epinephrine, isometheptene, midodrine, ergotamine tartrate, methylergonovine, lisuride, bromocriptine, methergine, naphazoline Immunossupressants/immunomodulators: FK‐506, cyclophosphamide, interferon, fingolimod, etanercept, tocilizumab, etoricoxib, ustekinumab, adalidumab, galcanezumab (CGRP‐antagonist), ciclosporin, mycophenolic acid Hormonal: oral‐contraceptive pills, ovarian stimulation, levonorgestrel intrauterine device, anastrozole, leuprorelin Other: indomethacin, phenytoin intoxication, hydroxycut, erythropoietin, intravenous immune globulin, nicotine patches, red blood cell transfusions, Ma Huang (ephedra), khat leaves, atovaquone, isoflavones, licorice, caffeine withdrawal, eucalyptus, ethylene oxide exposure, tetrodotoxin, oleoresin capsicum (pepper spray), snakebite/anti‐venom
Procedures	Post‐carotid endarterectomy, nasal sinus surgery, carotid stenting, open neurosurgical procedures, tonsillectomy, neck surgery, repetitive transcranial magnetic stimulation, dural puncture
Vascular pathologies	Unruptured saccular cerebral aneurysm, carotid and vertebral artery dissection, spinal subdural hematoma, cerebral venous sinus thrombosis, carotid glomus tumor, fibromuscular dysplasia, carotid web
Other medical conditions	Takotsubo cardiomyopathy, transient global amnesia, COVID‐19 infection, Chikungunya infection, COVID‐19 vaccination, Leigh syndrome, hypercalcemia, porphyria, hemolytic uremic syndrome, thrombotic thrombocytopenic purpura, elevated anti‐phospholipid antibodies, thyrotoxicosis, pheochromocytoma, bronchial carcinoid tumor, head trauma

Abbreviations: CGRP, calcitonin gene‐related peptide; COVID‐19, coronavirus disease 2019; FK‐506, tacrolimus; HELP, hemolysis, elevated liver enzymes, and low platelet count; LSD, lysergic acid diethylamide; PACNS, primary angiitis of the central nervous system; SNRI, serotonin noradrenaline‐reuptake inhibitors; SSRI, selective serotonin‐reuptake inhibitors; TCH, thunderclap headaches.

*Source*: Adapted from Singhal et al.^1^

### Vasoactive medications

Serotonergic and sympathomimetic drugs appear to be a common denominator in RCVS and rank among its most frequent triggers.^1^, ^3^, ^19^, ^20^, ^40^ Patients may experience TCH either upon first exposure to a drug or after prolonged use of one or multiple substances, at either therapeutic or excessive doses. Additionally, exposure to multiple vasoactive substances seems to be more common in men,^19^ and growing evidence suggests that serotonergic mechanisms play a more prominent role in RCVS than adrenergic pathways.^41^ This is supported by the high prevalence of migraine, depression, and anxiety among patients with RCVS, in contrast to the lower prevalence of sympathomimetic agent use. Furthermore, the distribution of serotonin receptors in the cerebral vasculature is believed to contribute to vasoconstriction and edema formation.^1^, ^19^, ^42^ More recently, a new class of medications, including anti‐calcitonin gene‐related peptide (CGRP) monoclonal antibodies, has been implicated in cerebral vasoconstriction, potentially mimicking RCVS.^43^

### Illicit drugs

Recently, a systematic review of the literature evaluated the relationship between RCVS and illicit drug use.^44^ The authors identified cannabis as the most commonly implicated substance, whereas evidence linking other drugs, such as cocaine, amphetamines, ecstasy, and khat, as isolated precipitating factors remains limited. Associations between marijuana and other drugs, including those derived from chloroform and ether, have been documented.^44^, ^45^ Regarding cocaine, evidence suggests that the vasoconstriction it induces primarily affects the intracranial carotid arteries rather than the arteries of the circle of Willis and their branches.^10^ However, establishing a direct causal link between a specific substance and RCVS is limited by poly‐drug use, drug adulteration, inconsistent diagnostic criteria, and the lack of prospective studies.^44^

### Pregnancy and postpartum

Since its initial descriptions, the association between the postpartum period, reversible leukoencephalopathy, and vasoconstriction syndromes has been recognized. Although often classified under RCVS, the prognosis can vary, with reports of rare but fatal cases.^43^ Compared with nonpregnant patients, postpartum cases had less exposure to vasoconstrictive illicit drugs.^31^ Most postpartum cases of RCVS begin within the first week after delivery, frequently complicated by conditions such as eclampsia or HELLP syndrome (hemolysis, elevated liver enzymes, and low platelet count); however, it can also occur in otherwise normal pregnancies.^46^

Regarding the relationship with pregnancy‐induced hypertension (PIH) syndromes, hypertension during pregnancy or the puerperium was present in 61% of postpartum RCVS cases, and PRES was reported in 54%.^47^ One cohort found radiographic evidence of PRES on magnetic resonance imaging (MRI) in 46 of 47 patients with eclampsia, consistent with the high imaging yield of PRES in obstetric neurologic emergencies.^48^ Moreover, prospective hemodynamic data in preeclampsia show increased anterior circulation velocities at diagnosis and on postpartum days 1 and 7, Lindegaard index–defined vasoconstriction in about one‐third of patients, and normalization by day 30, paralleling the reversibility seen in RCVS.^49^ Mechanistically, a shared substrate of acute hypertension, endothelial dysfunction, and transient failure of cerebrovascular autoregulation likely underlies the overlap among RCVS, PRES, and PIH; angiogenic imbalance involving placental growth factor and soluble fms‐like tyrosine kinase‐1, known correlates of preeclampsia, may also contribute to triggering RCVS.^50^ Even so, because classical obstetric features such as proteinuria, peripheral edema, and hepatic involvement are uncommon in RCVS outside the postpartum context, it is most precise to consider these as distinct entities that frequently coexist and share convergent mechanisms within an overlapping pathophysiological framework.^51^

Additionally, at least one‐third of postpartum cases involve exposure to vasoconstrictors used for epidural anesthesia, postpartum hemorrhage, inhibiting lactation, or treating depression.^46^, ^52^ Similar to the relatively high prevalence of postpartum RCVS, hemorrhagic complications are somewhat more frequent in this setting, with intracerebral hemorrhage as the most common presentation. Additionally, factors such as female sex, a history of migraine, and age over 45 years appear to be associated with an increased risk of the hemorrhagic form of the disease during the postpartum period.^32^

### Physiologic stressors

Physiological situations such as exertion, sexual intercourse, bathing, high altitude, straining during defecation, and coughing have been reported as potential triggers of TCH and RCVS. The likely underlying mechanism involves activation of sympathetic pathways and a surge in catecholamines.^39^, ^53^, ^54^, ^55^ Ducros and Wolff described a patient who experienced TCH while bending down to place flowers on her husband's grave during his funeral, suggesting that grief may act as a stress‐related trigger via catecholamine release. Additionally, other intense emotional states, such as fear and anger, have also been reported as potential triggers of RCVS.^39^, ^53^

### Miscellaneous and others

Various medical conditions have been identified as potential triggers of RCVS. Surgical procedures, neoplasms, metabolic disturbances, vascular structural abnormalities, and multisystemic diseases including autoimmune disorders and infections have all been implicated.^18^, ^19^, ^39^, ^56^, ^57^ However, the extent to which each of these factors directly contributes to the development of cerebral vasoconstriction remains uncertain.

Regarding structural abnormalities, RCVS has been associated with unruptured aneurysms, and we have described a yet unclear relationship between RCVS and carotid web, an intimal variant of fibromuscular dysplasia.^22^, ^28^, ^58^ Furthermore, the proportion of patients with RCVS presenting with cervical or vertebral artery dissections is considerable, affecting approximately 8% in French cohorts, suggesting a nonincidental association.^20^, ^28^, ^59^ Additionally, we report an association between RCVS, extracranial dissection, and coronavirus disease 2019 (COVID‐19) infection, suggesting that viral infection and its systemic effects may act as independent triggers for both vascular pathologies.^59^ In addition to COVID‐19, we documented a case of RCVS induced by the chikungunya virus, further supporting the concept of virus‐triggered RCVS.^60^

## OVERLAP SYNDROMES

RCVS shares several clinical, radiological, and proposed pathophysiological mechanisms with other syndromes, including primary headaches, TGA, and PRES.^1^

Primary TCH necessitates the exclusion of all potential secondary causes, with diagnostic criteria outlined in the International Classification of Headache Disorders, 3rd edition.^61^ However, evidence has challenged the notion of this syndrome as a distinct primary condition because vasoconstriction may not be detectable in the early stages in up to 20% of patients with confirmed RCVS, requiring a second imaging examination for diagnosis.^19^ Furthermore, a study by Chen et al. found no evidence of vasoconstriction in approximately 60% of patients presenting with TCH.^62^ Aside from the triggers of defecation and exertion, no significant differences in clinical presentation or rates of ischemia were observed between patients with and without vasoconstriction. These data indicate that primary TCH and RCVS may reflect a spectrum of the same disorder, leading to the introduction of the term *pure cephalalgic form of RCVS*.^20^, ^62^

TGA is marked by a sudden onset of both anterograde and retrograde amnesia, lasting up to 24 h, and is often accompanied by transient hyperintense MRI diffusion lesions in the hippocampal Cornu Ammonis 1 regions. This condition primarily affects individuals aged 50 to 70 years, with triggers documented in 50% to 90% of cases, including strenuous physical exertion, emotional or psychological stress, medical procedures, sexual intercourse, and Valsalva maneuvers, all of which are also associated with RCVS.^39^, ^63^ The first documented association between TGA and RCVS emerged in 2017, and subsequent reports have suggested a link between the two conditions.^39^, ^64^ However, although headache is the most common symptom in TGA, affecting approximately 40% of patients, its features tend to be nonspecific, contrasting with RCVS, where TCHs are present in the majority of cases. Moreover, another condition with shared characteristics is Takotsubo cardiomyopathy (TKC), which is characterized by transient left ventricular dysfunction that mimics acute coronary syndrome despite normal coronary arteries. This condition predominantly affects women aged 65 to 70 years and is frequently triggered by emotional or physical stress, with such triggers present in the majority of cases.^63^, ^65^ Thus, evidence suggests that TKC and RCVS may share pathophysiological mechanisms involving sympathetic overactivity and catecholamine excess.^66^ Notably, there is a documented instance of RCVS occurring sequentially with TKC, along with cases of TGA manifesting concurrently with TKC. Additionally, one patient with RCVS and TGA had a history of TKC 5 years earlier, suggesting a potential link among these conditions.^65^, ^66^

PRES is an acute neurological condition characterized by seizures, focal neurological deficits, headaches, and visual disturbances. MRI findings typically show bilateral hemispheric hyperintensities on T_2_‐weighted and fluid‐attenuated inversion recovery (FLAIR) images, with elevated apparent diffusion coefficient values. These hyperintensities often involve the cortex but are more prevalent in the subcortical and deep white matter regions, and vasogenic edema typically resolves within a few days.^67^ Reversible brain edema occurs in 8%–38% of cases of RCVS, and multifocal cerebral vasoconstriction is observed in up to 85% of patients with PRES. In terms of pathophysiology, both syndromes are associated with blood flow dysregulation and endothelial dysfunction, leading to the breakdown of the blood–brain barrier (BBB) and vasogenic edema, underscoring their frequent association.^19^, ^20^, ^28^, ^42^ Although they share clinical features and risk factors, PRES seems to be more closely associated with conditions like acute hypertension, renal failure, cytotoxic drug use, autoimmune disorders, and post‐transplantation scenarios.^67^ Moreover, whereas complications in RCVS are mainly hemorrhagic (most commonly cSAH), in PRES, ischemic and hemorrhagic events are equally common, with subarachnoid and intraparenchymal hemorrhages occurring in similar proportions.^68^ Thus, although RCVS can occur without vasogenic edema and PRES without vasoconstriction, shared pathophysiological mechanisms and possibly a common origin may explain the association between these two entities.^69^, ^70^

## PATHOPHYSIOLOGY

The pathophysiology of RCVS is not yet fully understood, but it is believed that dysfunction in the regulation of cerebral vascular tone and the impairment of the BBB play crucial roles in the disease's development, helping to explain some of its clinical and radiological manifestations.^71^ Although the origins of RCVS are heterogeneous, the underlying mechanisms are likely multifactorial. In addition to BBB disruption, other key factors include genetic predisposition, endothelial dysfunction, oxidative stress, and sympathetic hyperactivity, although the specific molecular mechanisms have not been fully elucidated.^72^

Dysregulation of cerebral vascular tone is a key factor in the pathophysiology of RCVS.^73^ The release of neurotransmitters such as norepinephrine and neuropeptide Y from sympathetic nerve endings may induce vasoconstriction, although the precise mechanism remains unclear and could either be an autoregulatory response or a result of a sudden increase in sympathetic activity.^74^ Additionally, the cerebral microcirculation is regulated by perivascular nerves that control vessel tone and vascular autoregulation.^74^ Sudden and unpredictable fluctuations in vascular tone, often driven by excessive sympathetic discharge, can result in cerebral vasoconstriction and, to a lesser extent, vasodilation.^72^, ^75^ Studies indicate that cerebrovascular reactivity is impaired during the acute phase of RCVS but may recover upon remission, suggesting a role for autoregulatory failure, oxidative stress, and endothelial dysfunction.^76^ These findings underscore the critical role of vascular tone dysregulation in RCVS pathogenesis and its interplay with other proposed mechanisms. Further molecular investigations may offer deeper insights into the underlying pathophysiology.

Sympathetic hyperactivity has been identified as a key factor in the pathogenesis of RCVS, with clinical evidence suggesting its association with the condition, as seen in cases of pheochromocytoma, following the use of sympathomimetic substances, or during acute hypertensive crises.^18^, ^39^ Heart rate variability studies have shown heightened sympathetic activity and reduced parasympathetic modulation during the acute phase of the disease.^75^ This autonomic dysregulation, which only partially recovers during remission, suggests a predisposition to a sympathetic‐parasympathetic imbalance in patients with RCVS. In parallel, endothelial dysfunction plays a critical role in disease pathophysiology given its fundamental role in regulating cerebral vascular tone.^75^ Patients with RCVS exhibit impaired endothelial‐dependent vasodilation, as demonstrated by a diminished response to hypercapnia.^77^ Furthermore, individuals with RCVS exhibit a reduced number of circulating endothelial progenitor cells (CD34 + KDR+), particularly those with more severe vasoconstriction.^78^ Endothelial dysfunction is further emphasized by its association with overlapping syndromes such as PRES, as well as vascular tone dysregulation, hypertensive crises, and exposure to immunosuppressive or cytotoxic agents.^73^, ^75^

Oxidative stress exhibits a complex interaction with endothelial dysfunction and sympathetic hyperactivity in the regulation of vascular tone.^78^ Although its contribution to the pathogenesis of RCVS is plausible, there remains a paucity of studies on the topic. A prospective study found elevated levels of 8‐iso‐prostaglandin F2α (8‐iso‐PGF2α), a reliable marker of oxidative stress and potent vasoconstrictor, in the urine of patients with RCVS during the acute phase, with normalization during remission.^78^ Other metabolic studies confirm the presence of oxidative stress, identifying metabolic signatures associated with free radical scavenging processes and the metabolism of vitamins and minerals, highlighting the role of oxidative stress in the pathogenesis of RCVS and its interactions with other mechanisms, such as endothelial dysfunction and excessive sympathetic activity.^79^

Additionally, BBB dysfunction has been suggested as a key factor in RCVS. When cerebral autoregulation is overloaded, the integrity of the BBB can be compromised, leading to ischemic or hemorrhagic complications. Research has indicated that up to 70% of patients with RCVS show signs of BBB disruption, with hemorrhagic complications or PRES occurring within the first few weeks of the disease.^80^ Even in patients without evidence of macroscopic disruption, the microscopic permeability of the BBB is increased, and molecules such as miR‐130a‐3p may serve as biomarkers of BBB dysfunction, modulating its permeability in response to hemodynamic changes.^81^, ^82^ Because cerebral vascular function and BBB permeability are strongly influenced by estrogen and progesterone, these sex hormones are believed to play a role in RCVS pathogenesis, although direct evidence is lacking.^83^ Some case reports have linked RCVS onset to acute hormonal changes, use of oral contraceptives, levonorgestrel intrauterine devices, and ovarian stimulation medication;^21^, ^84^, ^85^, ^86^ however, establishing a causal relationship based on these cases is challenging. A large retrospective study found no significant differences between premenopausal and postmenopausal women, or between those with and without hysterectomy.^31^ Thus, the precise role of hormonal imbalance in triggering or modulating RCVS remains unclear.

In relation to the TCH in RCVS, its underlying mechanism remains unclear, although the involvement of distal arterioles may play a critical role. The severe pain observed in the initial weeks of the condition, prior to the peak vasoconstriction, suggests that the activation of the trigeminovascular pathway, which innervates the cerebral vessels, is more influential than the constriction of larger arteries.^82^ During the course of the disease, an exaggerated release of calcitonin gene‐related peptide may activate trigeminal nociceptors, contributing to the pain.^74^ Furthermore, microRNAs like let‐7a‐5p and let‐7b‐5p, which are elevated in both RCVS and migraine, may be linked to the CGRP‐dependent trigeminovascular reflex, suggesting common mechanisms between these conditions.^81^

Although no clear hereditary pattern or definitive susceptibility gene has been identified, findings such as potential recurrence, persistent autonomic dysfunction, and associations with monogenic disorders (e.g., Ehlers–Danlos) suggest a genetic link in RCVS.^72^, ^87^, ^88^ Consistent with this, one study showed that the brain‐derived neurotrophic factor Val66Met polymorphism is associated with more severe clinical presentations.^89^ Furthermore, an association between RCVS and Leigh syndrome has been described, and recently, two intrafamilial cases in two different families have reinforced the hypothesis of a genetic component.^90^, ^91^ However, no association was found between RCVS and *ring finger protein 213*, a susceptibility gene for moyamoya disease and for intracranial arterial stenosis and dissection.^92^ Thus, given the complexity of the disease and the involvement of environmental factors, RCVS is likely multifactorial and modulated by multiple genes with small effect sizes.^72^

**FIGURE 2:**
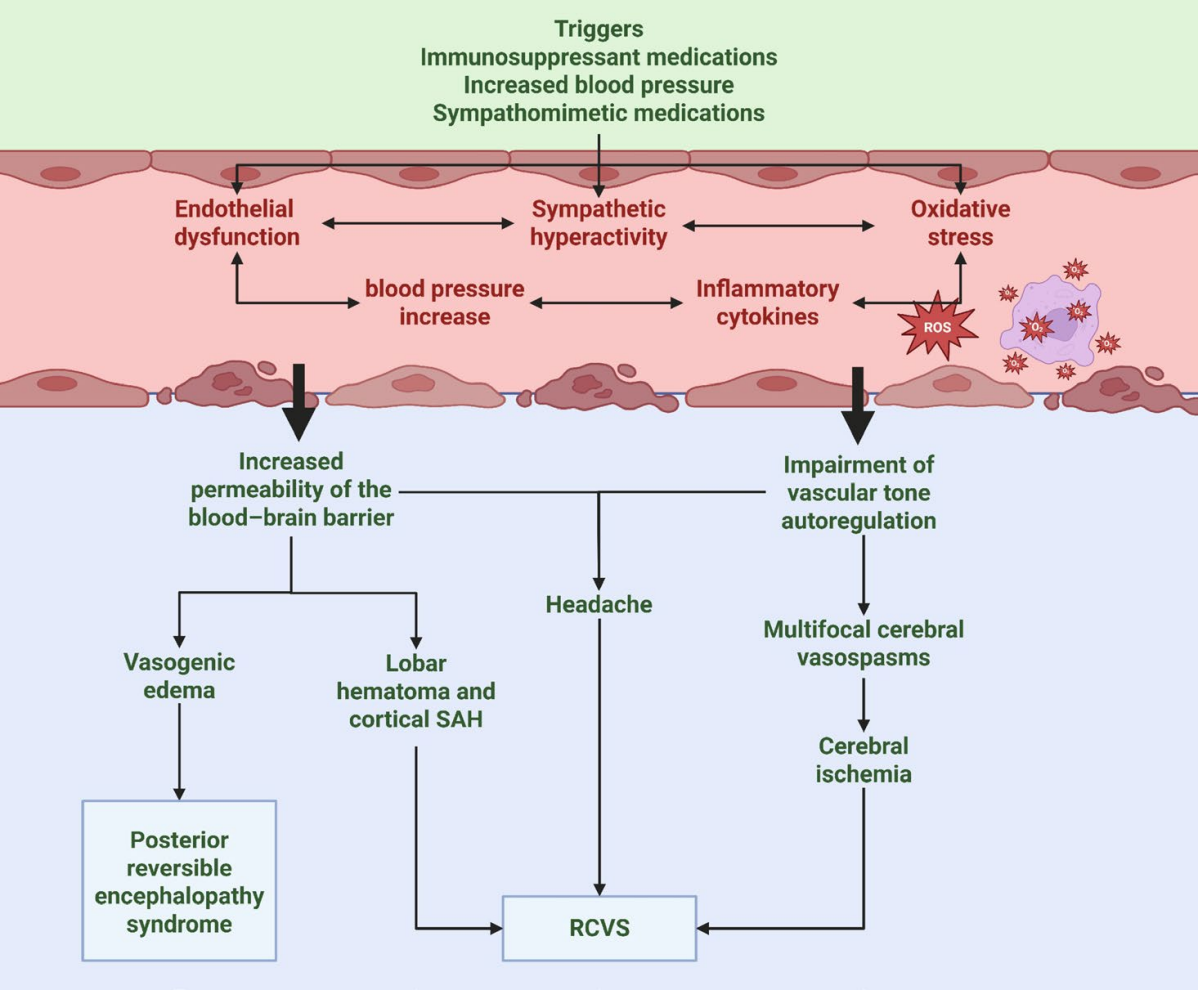
Pathophysiology of RCVS proposed. Pathophysiology model adapted from Chen and Wang in which precipitating factors contribute to endothelial dysfunction, sympathetic overactivity and oxidative stress, especially in genetically predisposed individuals.^72^ [Color figure can be viewed at wileyonlinelibrary.com]

Chen and Wang proposed a model for RCVS pathophysiology, suggesting that genetically predisposed individuals are more vulnerable to triggers.^72^ Sympathetic activation leads to the release of vasoconstrictors, disrupting cerebral vascular tone, causing distal arteriolar dilation—likely due to an exaggerated trigeminovascular reflex—and inducing severe pain. Increased central pulsatile flow and elevated blood pressure impair the neurovascular unit, increasing BBB permeability and contributing to vascular tone dysfunction and headache.^71^, ^72^ Persistent sympathetic activation causes endothelial dysfunction and oxidative stress, perpetuating vascular dysregulation. Endothelial repair may be exhausted, and vasoactive metabolites like 8‐iso‐PGF2α and leaked plasma components worsen vasoconstriction.^72^, ^78^ Vascular wall inflammation may occur with prolonged vasoconstriction. Shear stress and vascular tone dysregulation lead to increased microRNA expression (e.g., miR‐130a‐3p, miR‐130b‐3p), contributing to acute pain and BBB disruption. BBB breakdown and increased central pulsatile flow may cause white matter hyperintensities (WMHs) and early complications like PRES, subarachnoid, or intracerebral hemorrhage.^72^, ^81^ In contrast, vasoconstriction of major arteries may cause hypoperfusion, worsening WMHs, or leading to ischemic complications and worsening vasogenic edema. However, some mechanisms primarily apply to idiopathic RCVS, and further studies are needed for secondary RCVS cases.^72^ Figure 2 summarizes the proposed pathophysiological mechanisms.

## CLINICAL FEATURES

### General aspects

The hallmark symptom of this condition is the abrupt onset of a severe headache, reaching its peak intensity in under 1 min (termed *thunderclap headache*). In prior cohorts, TCH was reported in 95% to 100% of patients with RCVS; in 76% to 85% of patients, it may be the sole clinical manifestation.^39^, ^93^, ^94^, ^95^ It begins hyperacutely, with “worst‐ever” pain that peaks within seconds and can provoke screaming, agitation, panic, or fear of dying; sympathetic overactivity is common; and about one‐third of patients have surges in blood pressure.^19^, ^96^ The pain is usually bilateral, often starting occipitally and becoming diffuse within seconds, although unilateral attacks occur in up to 19%. Beyond headaches, patients frequently report symptoms such as nausea, vomiting, photophobia, and phonophobia.^94^, ^97^, ^98^, ^99^ Furthermore, in contrast to aneurysmal subarachnoid hemorrhage (aSAH), RCVS attacks are typically short‐lived, with an average duration of 1 to 3 h.^19^ Headache episodes can recur in 94% to 100% of patients within a span of 1 to 3 weeks^100^ and may be precipitated or aggravated by Valsalva maneuvers.^93^, ^100^, ^101^ Additionally, less intense headaches of a persistent nature may occur between episodes of TCHs.^102^

Recent data indicate that up to 30% of patients may present without TCH.^103^ Non‐TCHs in RCVS are heterogeneous, with onset that can be rapid within a few minutes up to 10 min or more gradual; in some cases, characterization is not possible because consciousness is impaired.^46^, ^104^ Intensity ranges from mild to severe; location can be diffuse or focal in the occipital, frontal, frontotemporal, temporal, or vertex regions; laterality may be unilateral or bilateral; and a pounding quality can occur.^46^, ^98^ Duration varies from minutes to days, although most episodes are short‐lived and align with the temporal profile typically observed in RCVS with TCH.^98^, ^105^ Moreover, the absence of TCH is associated with increased risk of cerebral complications, including ischemic stroke and intracerebral hemorrhage.^40^, ^103^, ^106^

Focal neurological deficits are also common, affecting approximately 8% to 43% of cases,^107^ and these can include encephalopathy, visual disturbances, dysarthria, aphasia, ataxia, seizures, and focal numbness or weakness.^101^, ^108^ Rarely, Balint syndrome can occur as a consequence.^109^ The majority of patients exhibit hyperreflexia and mild tremor, which may indicate increased serotonergic activity.^1^ Blood pressure fluctuations are common at the onset of symptoms, likely due to the combination of intense headaches and cerebrovascular autoregulation in the context of cerebral vasoconstriction.^19^ Recently, a spinal cord syndrome presenting with quadriparesis due to a high‐cervical spinal cord infarct has been described in association with RCVS.^110^ Moreover, symptoms such as chest and abdominal pain attributable to concurrent extracranial vasoconstriction have been reported.^111^

Although the disease course is traditionally considered monophasic and benign, data indicate that approximately one‐third of patients experience clinical deterioration within the first 14 days after diagnosis, with permanent neurological deficits occurring in 40% of these cases, and in some instances, fatalities, particularly in postpartum women. This unfavorable clinical course is primarily associated with the presence of infarctions in brain parenchyma imaging studies.^52^

### Neuropsychological aspects

Despite significant advances in understanding RCVS in recent years, its neuropsychological aspects remain largely overlooked. Until last year, only a single report had addressed this issue, describing a young patient who, 16 months post‐RCVS, exhibited deficits in autobiographical memory, verbal and nonverbal learning, delayed recall, cognitive flexibility, and abstraction, with milder impairments in attention span and processing speed.^56^

To further investigate this, we conducted a case series of highly educated patients with RCVS who underwent neuropsychological evaluation, revealing that cognitive impairment is more common than previously recognized and may contribute to persistent post‐RCVS symptoms. Most patients demonstrated deficits in attention and executive function, whereas visuoconstructive impairments were less frequent. In contrast, nonverbal memory and language functions remained relatively preserved. Notably, all patients performed well on the Mini Mental Status Exam, underscoring the need for comprehensive neuropsychological assessments in those with cognitive complaints following RCVS.^112^ However, these findings are preliminary, and further studies with larger cohorts and more in‐depth neuropsychological evaluations are necessary.

## NEUROIMAGING

**FIGURE 3:**
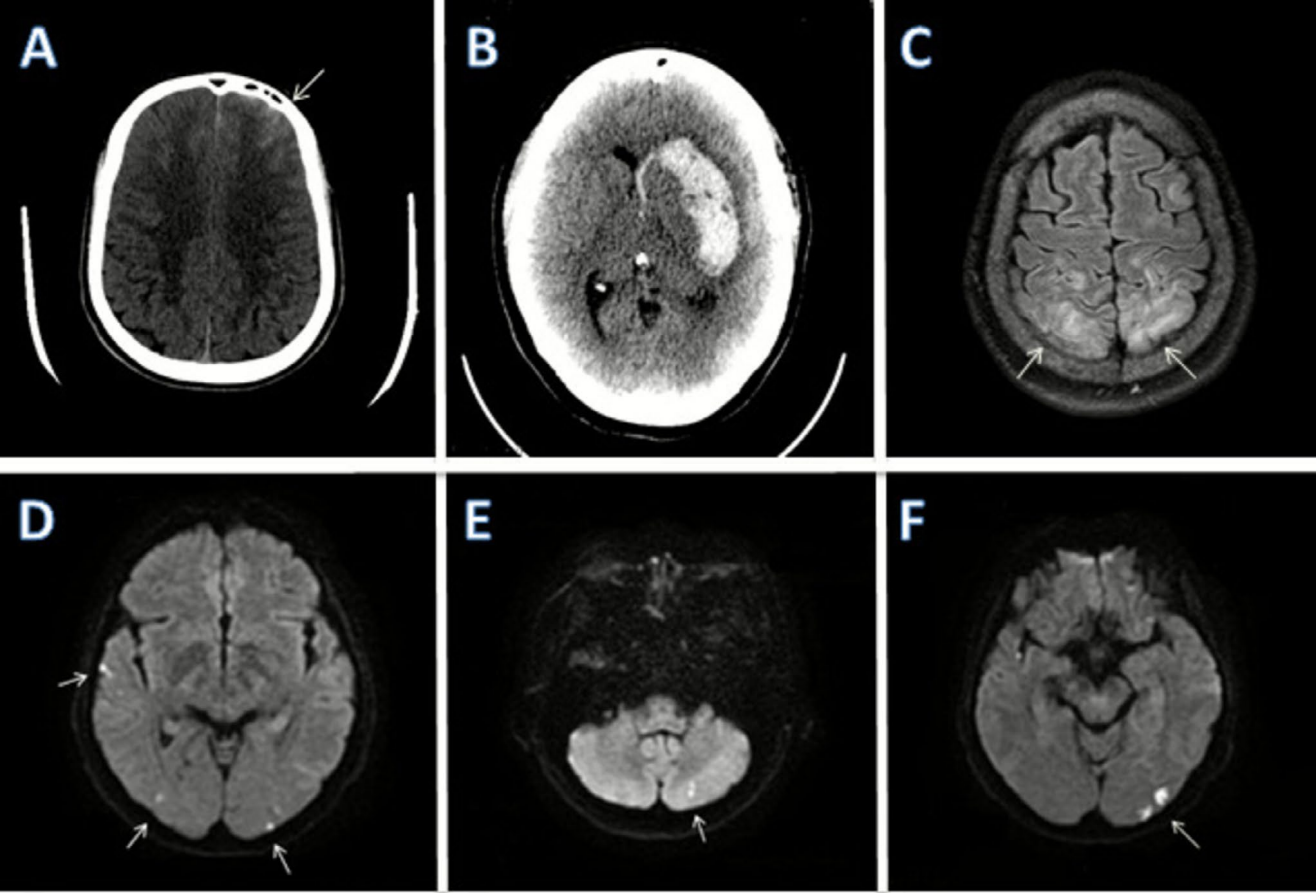
Typical parenchymal findings in patients with RCVS. Brain CT showing a left frontal convexity subarachnoid hemorrhage (A) and a left basal nuclei hematoma with ventricular extension (white arrows) (B). Brain MRI on the FLAIR sequence showing bilateral hyperintensity in the posterior territories, suggestive of vasogenic edema (C). The DWI sequence shows multiple diffusion restriction foci in cortical, subcortical, and watershed areas (white arrows), observed in the right temporal and bilateral occipital lobes (D), the left cerebellar hemisphere (E), and the left occipital lobe (F). CT, computed tomography; DWI, diffusion‐weighted imaging; FLAIR, fluid‐attenuated inversion recovery; MRI, magnetic resonance imaging. [Color figure can be viewed at wileyonlinelibrary.com]

**FIGURE 4:**
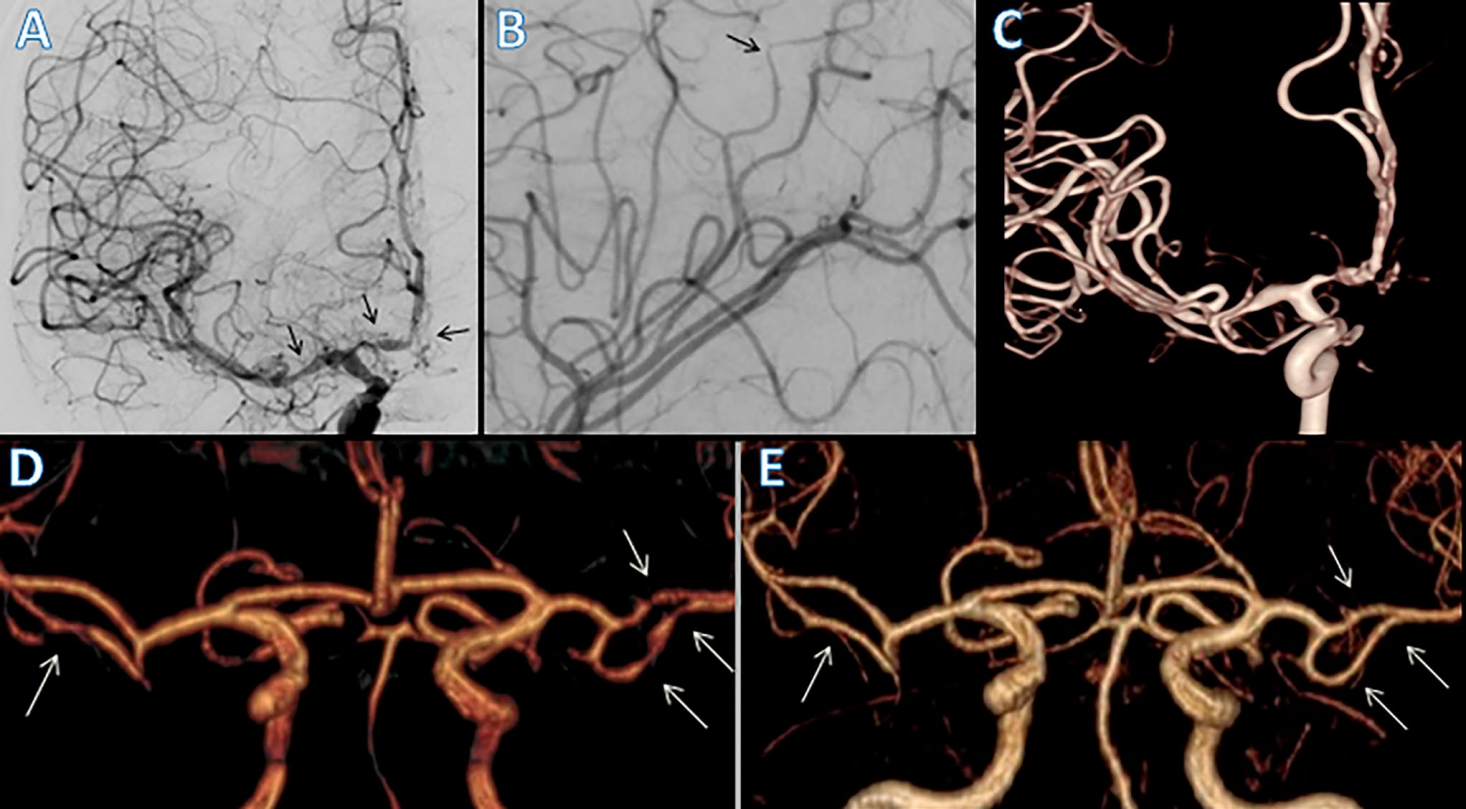
Typical vascular findings in patients with RCVS. DSA in an anteroposterior view showing proximal segmental vasoconstriction in the M1 segment of the right MCA and the A1 and A2 segments of the right ACA (A). Distal subtle segmental narrowing (black arrow), followed by a normal appearance in the left distal branch of the middle cerebral artery compatible with vasoconstriction (B). Tridimensional rotational angiography 3D‐RA demonstrating areas of segmental and multifocal vasoconstriction in cerebral arteries (C). MRA showing multiple areas of segmental narrowing (white arrows) in bilateral middle cerebral arteries (D). MRA showing reversal of vasoconstriction findings after 100 days, consistent with reversible cerebral vasoconstriction syndrome (E). 3D‐RA, three‐dimensional rotational angiography; ACA, anterior cerebral artery; DSA, digital subtraction angiography; MCA, middle cerebral artery; MRA, magnetic resonance angiography. [Color figure can be viewed at wileyonlinelibrary.com]

Neuroimaging plays a pivotal role in diagnosis, monitoring disease evolution, and differentiating RCVS from other vasculopathies such as primary angiitis of the central nervous system (PACNS), aSAH, and intracranial atherosclerosis.^1^, ^27^, ^113^ Figures 3 and 4 summarize the radiological findings of RCVS.

### Parenchymal findings on non‐contrast computed tomography

Non‐contrast computed tomography (CT) is often the first‐line imaging modality in patients presenting with TCH. However, it is normal in more than 50% of cases.^29^ When abnormalities are detected, they typically include cSHA, intracerebral hemorrhage, and hypodensities in watershed territories suggestive of early ischemic infarcts.^19^, ^29^, ^113^ In addition, a hyperdense vessel sign has been described in the context of RCVS.^114^

cSAH is the most frequent hemorrhagic manifestation, observed in 20%–38%^28^, ^29^, ^101^, ^115^ of cases. Unlike aSAH, which predominantly affects the basal cisterns, cSAH in RCVS primarily involves the cortical sulci. Intracerebral hemorrhage occurs in a smaller subset of patients, ranging from 7% to 34%, and predominantly affects lobar regions rather than deep gray matter.^20^, ^116^ Additionally, early ischemic infarcts may present as subtle hypodensities in watershed territories, although these changes can be undetectable in the hyperacute phase.^22^, ^116^ Vasoconstriction typically follows a centripetal course in RCVS, with hemorrhagic features associated with distal vasoconstriction generally appearing within the first few days of the disease, whereas ischemic changes emerge around 1 week later.^117^

### Parenchymal findings on MRI

MRI is more sensitive than CT for detecting early parenchymal changes in RCVS, particularly ischemic and vasogenic complications.^118^ Diffusion‐weighted imaging is crucial for identifying ischemic infarcts, which occur in approximately 30%–39% of cases.^27^, ^116^ These infarcts predominantly affect watershed areas or the posterior circulation, reflecting impaired autoregulation, and are characterized by restricted diffusion.^27^, ^29^

FLAIR imaging reveals additional key features, including hyperintense sulcal signals indicating cSAH and vasogenic edema with a PRES‐like pattern.^119^ This vasogenic edema occurs in 28% of patients and is typically bilateral and symmetrical, predominantly involving the parieto‐occipital and frontal lobes, although brainstem and deep gray matter involvement can also occur.^108^, ^113^ Another distinctive finding in FLAIR imaging is the “dot sign,” described as small hyperintense dots in the sulci, which may indicate slow flow or small‐vessel thrombosis.^27^, ^116^, ^119^

### Vascular imaging findings: Computed tomography angiography, magnetic resonance angiography, and digital subtraction angiography

#### Computed tomography angiography and magnetic resonance angiography

Computed tomography angiography (CTA) and magnetic resonance angiography (MRA) offer noninvasive assessment of cerebral vasculature but may appear normal in the early stages due to the dynamic nature of vasoconstriction.^19^, ^28^, ^29^ Consequently, repeat imaging at 7–14 days is often necessary to capture the evolution of arterial changes.^105^

The hallmark angiographic feature of RCVS is the “string of beads” or “sausage on a string” appearance, characterized by alternating areas of constriction and dilation.^27^ This pattern primarily affects the middle cerebral artery, anterior cerebral artery, and posterior cerebral artery.^120^ Vasoconstriction is predominantly detected in the proximal M2, A2, and P2 segments, but distal branches can also be affected.^1^, ^113^, ^116^ Although CTA and MRA are valuable screening tools, they may underestimate distal involvement, necessitating DSA in uncertain cases.^20^, ^116^

In a retrospective cohort, serial MRA showed that vasoconstriction in RCVS often follows a centripetal pattern, beginning in distal segments such as M2, P2, and A2 and advancing toward proximal arteries. This pattern was identified in about half of patients and was associated with longer duration of TCHs and higher rates of infarction or cortical subarachnoid hemorrhage.^121^ Vasoconstriction typically peaks around the time headaches remit and does not progress thereafter, supporting repeated vascular imaging from symptom onset through remission.^117^ Even when early angiography is normal, subsequent MRA may reveal evolving disease, underscoring the value of serial imaging in suspected RCVS.^121^

#### Digital subtraction angiography

DSA remains the gold standard for diagnosing RCVS, particularly in detecting subtle or distal vasoconstriction that may be missed on CTA/MRA.^122^ The characteristic multifocal segmental stenoses seen on DSA typically resolve on follow‐up imaging within 12 weeks, distinguishing RCVS from chronic vasculopathies.^15^ Additionally, DSA can demonstrate dynamic vasospasm, occasionally reversible with intra‐arterial vasodilator administration such as calcium channel blockers.^37^, ^123^ However, DSA is an invasive procedure with potential complications, including arterial dissection, stroke, transient ischemic attacks, and contrast‐induced nephropathy. Therefore, careful patient selection and risk–benefit assessment are necessary.^113^, ^116^

### Emerging imaging techniques in RCVS diagnosis

Advancements in neuroimaging have led to the development of high‐resolution vessel wall imaging (VWI) and arterial spin labeling (ASL) perfusion MRI, providing deeper insights into the pathophysiology of RCVS and aiding in its differentiation from other vasculopathies.^113^

#### VWI

High‐resolution VWI using MRI allows for direct evaluation of intracranial arterial walls. In RCVS, VWI typically demonstrates an absence of arterial wall enhancement.^27^ However, emerging evidence indicates that this classic pattern is not universal. In a Taiwanese prospective cohort of 48 patients with RCVS, nearly half (45.8%) demonstrated some degree of vascular wall enhancement on T_1_‐FLAIR VWI; and in about one‐third of those patients, the enhancement persisted on follow‐up imaging months later.^120^ Thus, VWI findings should always be interpreted in a clinical context and not used alone for diagnosis.

Nevertheless, one of the most challenging applications of VWI is in differentiating RCVS from primary CNS vasculitis because their clinical presentations and angiographic findings often overlap.^124^ High‐resolution VWI offers crucial diagnostic clues. In a seminal study, Mandell et al. prospectively evaluated patients with multifocal arterial narrowing and found that RCVS was characterized by arterial wall thickening without enhancement, whereas vasculitis demonstrated concentric wall enhancement and persistent stenosis on follow‐up.^125^ These imaging patterns reflect their underlying pathology: transient vasoconstriction without inflammation in RCVS versus inflammatory wall infiltration in vasculitis. VWI can therefore help clinicians make timely treatment decisions.

#### ASL perfusion MRI

ASL MRI is an emerging perfusion imaging technique that can detect early‐stage hypoperfusion before anatomical stenoses become apparent.^126^ ASL findings in RCVS include decreased cerebral blood flow (CBF) in vasoconstricted territories, particularly in watershed areas, correlating with stroke risk. Notably, ASL can be used to monitor the progression of RCVS and evaluate treatment effectiveness because its perfusion deficits are reversible and parallel the resolution of vasoconstriction.^127^ In a five‐patient RCVS series, ASL was more sensitive than MRA for detecting hypoperfusion, with lower CBF in the first week followed by a subsequent increase. Moreover, regions affected by PRES or subarachnoid hemorrhage showed lower CBF than other areas, consistent with impaired autoregulation. Together, these findings highlight ASL as a useful adjunct for monitoring perfusion deficits and disease dynamics in RCVS.^128^

Neuroimaging is indispensable for the diagnosis, monitoring, and prognostication of RCVS. The characteristic “string of beads” pattern on angiography, parenchymal ischemic, or hemorrhagic changes as well as emerging techniques such as VWI and ASL perfusion MRI provide a comprehensive framework for understanding this dynamic syndrome. Given the reversible nature of vasoconstriction, repeat imaging is useful to confirm resolution and distinguish RCVS from mimicking conditions. Continued advancements in imaging modalities will likely enhance diagnostic accuracy in the future.

## DIAGNOSTIC APPROACH AND DIFFERENTIAL DIAGNOSIS

The evaluation of patients with RCVS can be complex, primarily because this condition presents clinical and radiological findings that significantly overlap with other diseases. From a clinical standpoint, TCH is typically the first hallmark that prompts further investigation.

Although TCH is characteristic of RCVS, it is not specific and can occur in patients with other potentially serious conditions, such as aneurysmal rupture, arterial dissections, pituitary apoplexy, meningitis, cerebral venous thrombosis (CVT), and CNS vasculitis, as well as in primary headache disorders including orgasmic headache, Valsalva‐associated headache, exercise‐induced headache, and primary TCH. Clinical evaluation, combined with neuroimaging including vessel studies (arterial and venous) and cerebrospinal fluid (CSF) analysis, is generally sufficient to differentiate the main causes of potentially severe TCH.^39^, ^129^

Clinical scoring systems, such as the RCVS‐TCH score, have recently been applied to estimate the likelihood of TCH being associated with RCVS (Table 1). This score integrates features commonly linked to RCVS, including recurrent TCH, female sex, and elevated blood pressure, to stratify risk. A score ≥7 differentiates RCVS from alternative diagnoses with 80% sensitivity and 97% specificity. Importantly, high RCVS‐TCH scores should not preclude a systematic evaluation for aSAH because the score was developed following rigorous exclusion of such cases.^129^

The RCVS2 score, also used in clinical practice, incorporates both clinical variables, such as TCH, prior exposure to vasoactive triggers, and female sex, and radiological/laboratory findings, including the absence of carotid stenoses and presence of subarachnoid hemorrhage (Table 2). This tool is particularly useful in distinguishing RCVS from other cerebral arteriopathies, such as PACNS.^36^ A score ≥5 demonstrates 99% specificity and 90% sensitivity for confirming RCVS, whereas scores ≤2 offer 100% specificity and 85% sensitivity for excluding the diagnosis. Scores between 3 and 4 yield intermediate diagnostic performance (86% specificity, 10% sensitivity).^36^ In patients with intermediate scores, the authors suggest a diagnostic approach that utilizes highly specific features of RCVS, including recurrent TCHs, normal imaging plus a vasoconstrictive trigger, and cortical subarachnoid hemorrhage.

**TABLE 2:** Scores for RCVS diagnosis.

RCVS–TCH score^a^	Value^d^	RCVS2 score^b^	Value^d^
Pattern of TCHs		Recurrent or single TCH	
Recurrent	2	Present	5
Single	0	Absent	0
Sex		Carotid artery (intracranial)	
Female	3	Affected	‐2
Male	0	Not affected	0
Triggering factor for TCH		Vasoconstrictive trigger	
Multiple	3	Present	3
Single	2	Absent	0
None	0		
BP surge^c^		Sex	
Present	4	Female	1
Absent	0	Male	0
		Subarachnoid hemorrhage	
		Present	1
		Absent	0

Abbreviations: BP, blood pressure; RCVS, reversible cerebral vasoconstriction syndrome; TCH, thunderclap headaches.

^a^ Cho et al.^129^

^b^ Rocha et al.^36^

^c^ BP surge defined as systolic BP of >160 mmHg during headache attacks or >30 mmHg from baseline.

^d^ RCVS‐TCH score ≥7 and RCVS2 score ≥5 suggest a diagnosis of RCVS.

Although these scoring systems provide structured diagnostic support in key RCVS scenarios, namely the evaluation of TCH and the differentiation from other cerebral vasculopathies, familiarity with their limitations remains essential. Several mimics may exhibit overlapping clinical and radiological features, underscoring the need for comprehensive diagnostic assessment beyond score‐based stratification.

In some cases aSAH can pose a diagnostic challenge when distinguishing it from RCVS. Both conditions share several features, such as TCH, subarachnoid hemorrhage, and intracranial stenoses (vasospasms) (Table 3). The high prevalence of unruptured aneurysms in the general population (3%–8%),^130^ particularly when associated with the clinical symptoms mentioned, may direct the diagnosis toward aSAH in patients with RCVS. Patients with SAH associated with RCVS (SAH‐RCVS) typically exhibit widespread arterial stenoses, but the SAH is usually confined to 1–3 sulcal spaces (cSAH). Conversely, patients with aSAH often present evidence of bleeding in multiple sulci, accompanied by more localized stenoses, which are typically situated near the bleeding sites.^131^ In a retrospective study, SAH‐RCVS was limited to 1–3 adjacent sulci and was unilaterally distributed in 83% of cases.^131^ Additionally, in aSAH, blood frequently fills the basal cisterns, whereas cortical SAH is the typical pattern observed in patients with RCVS.^131^ Although both conditions are classical causes of TCH, approximately 80% of patients with aSAH experience only a single episode,^130^ whereas 80%–100% of patients with RCVS experience 3–4 recurrent episodes over a few days.^132^ Thus, the recurrence of TCH is a critical factor in differentiating these conditions.

**TABLE 3:** Comparison of key clinical, imaging, and laboratory features of RCVS and its major differential diagnoses.

	RCVS	aSAH	Vasculitis of the central nervous system
Clinical findings			
Headache	TCH is common, and its recurrence in the first few days is a milestone of the disease.	TCH is common, but its recurrence is rare	Approximately 50% of patients have headache; only 6% have TCH^a^
Other neurological manifestations	Focal neurological deficits are secondary to cortical and border area strokes.	Intracranial hypertension syndrome and decreased level of consciousness are common	Rapidly progressive dementia
	Decreased level of consciousness and other focal, mainly cortical signs may occur in patients with superimposed PRES	Focal neurological deficits associated with delayed cerebral ischemia	Multiple focal neurological deficits secondary to infarcts, tumor‐like lesions, and intracranial hemorrhage
Radiological findings			
Cranial MR/CT	It may be normal for the first few days	CT may be normal (Fisher modified 0)	Multiple strokes at different ages adjacent to white matter disease
	Cortical SAH is usually restricted to 1–3 sulci. Usually small cortical infarcts and larger watershed infarcts	More diffuse bleeding with involvement of multiple cerebral sulci and basal cisterns	Intralesional foci of spontaneous bleeding. There may be leptomeningeal enhancement in some cases.
	Vasogenic edema of the parieto‐occipital and border zones compatible with PRES may be seen in one‐third of cases^b^		
Vascular study	Multifocal stenoses Stenoses are spontaneously reversible within 3 months	Aneurysm is usually identified	Arteries with irregular, notched, and eccentric stenoses and ectasias
Angio‐MRI and angio‐CT	Reversible stenoses with intra‐arterial infusion of milrionne and calcium channel blockers	More focal stenoses located closer to the bleeding site Reversible stenoses with intra‐arterial infusion of milrionne and calcium channel blockers	VWI demonstrates homogeneous circumferential involvement of vessels.Stenoses do not reverse with intraarterial infusion of milrinone or calcium channel blockers.
Cerebrospinal fluid	Usually normal. Elevated protein levels may be found. In rare cases, pleocytosis may be present.	Increased red blood cells, particularly crenated, xanthochromia, and increased protein. Cellularity is usually normal.	CSF is altered in 90% of cases. Pleocytosis, increased protein, and the presence of oligoclonal bands are typical findings.

Abbreviations: aSAH, aneurysmal subarachnoid hemorrhage; CSF, cerebrospinal fluid; CT, computed tomography; MR, magnetic resonance; PRES, posterior reversible encephalopathy syndrome; RCVS, reversible cerebral vasoconstriction syndrome; SAH, subarachnoid hemorrhage; TCH, thunderclap headaches; VWI, vessel wall imaging.

^a^ Singhal.^108^

^b^ Singhal et al.^29^

PACNS are conditions that are frequently confused with one another, having only been differentiated as specific entities in 1990.^20^ Similar to RCVS, patients with PACNS also present with headache; however, only 6% can be classified as TCH.^108^ In contrast to RCVS, PACNS is associated with poor outcomes in up to 40% of patients and a higher in‐hospital mortality based on Nationwide Inpatient Sample data (5% vs. 0.3%).^25^, ^133^ Focal neurological deficits are more common and accumulate over time,^124^ leading to the identification of multiple areas consistent with ischemic events at different ages on cranial MRI. These areas often exhibit extensive regions of surrounding white matter disease.^134^ Furthermore, it is not uncommon for patients with PACNS to experience cognitive decline, which is one of the classic causes of rapidly progressive dementia.^134^, ^135^ In other words, PACNS is a disease that exhibits a more indolent behavior compared to RCVS, which presents with hyperacute onset symptoms. The CSF of patients with PACNS is altered in 90% of cases, characterized by mild pleocytosis, increased protein levels, and the presence of oligoclonal bands, findings that are quite atypical in patients with RCVS.^20^, ^135^ Various different diseases are encompassed within PACNS; consequently, heterogeneous clinical and radiological findings may be observed. Generally, the most common forms of PACNS affect small vessels, thus not presenting with evident arterial stenoses.^134^ However, in subtypes where stenoses are found, and consequently the diagnostic dilemma with RCVS becomes relevant, the use of cranial MRI with the VWI sequence may be useful. The pattern most typically observed is scattered foci of smooth, homogeneous circumferential involvement of vessels.^135^ In cases of persistent diagnostic uncertainty accompanied by progressive clinical deterioration, invasive approaches, such as intra‐arterial infusion of milrinone or calcium channel blockers during cerebral angiography, may be attempted. These interventions can potentially induce reversal of arterial stenoses in RCVS, sometimes even affecting vessels contralateral to the side of administration.^123^, ^136^ In contrast, such reversal is generally not observed in primary CNS vasculitides.^37^

In clinical practice, the differentiation between these entities is of utmost importance because the immunosuppressive treatment with corticosteroids, frequently used in PACNS, is associated with worse outcomes in patients with RCVS.^29^ In scenarios in which differentiation is not possible solely based on clinical, radiological, and laboratory data, waiting a few days before determining an aggressive therapeutic approach may be the best option because patients with RCVS generally tend to stabilize and improve clinically, with complete reversal of vasoconstriction, whereas this does not occur in PACNS.^25^

**FIGURE 5:**
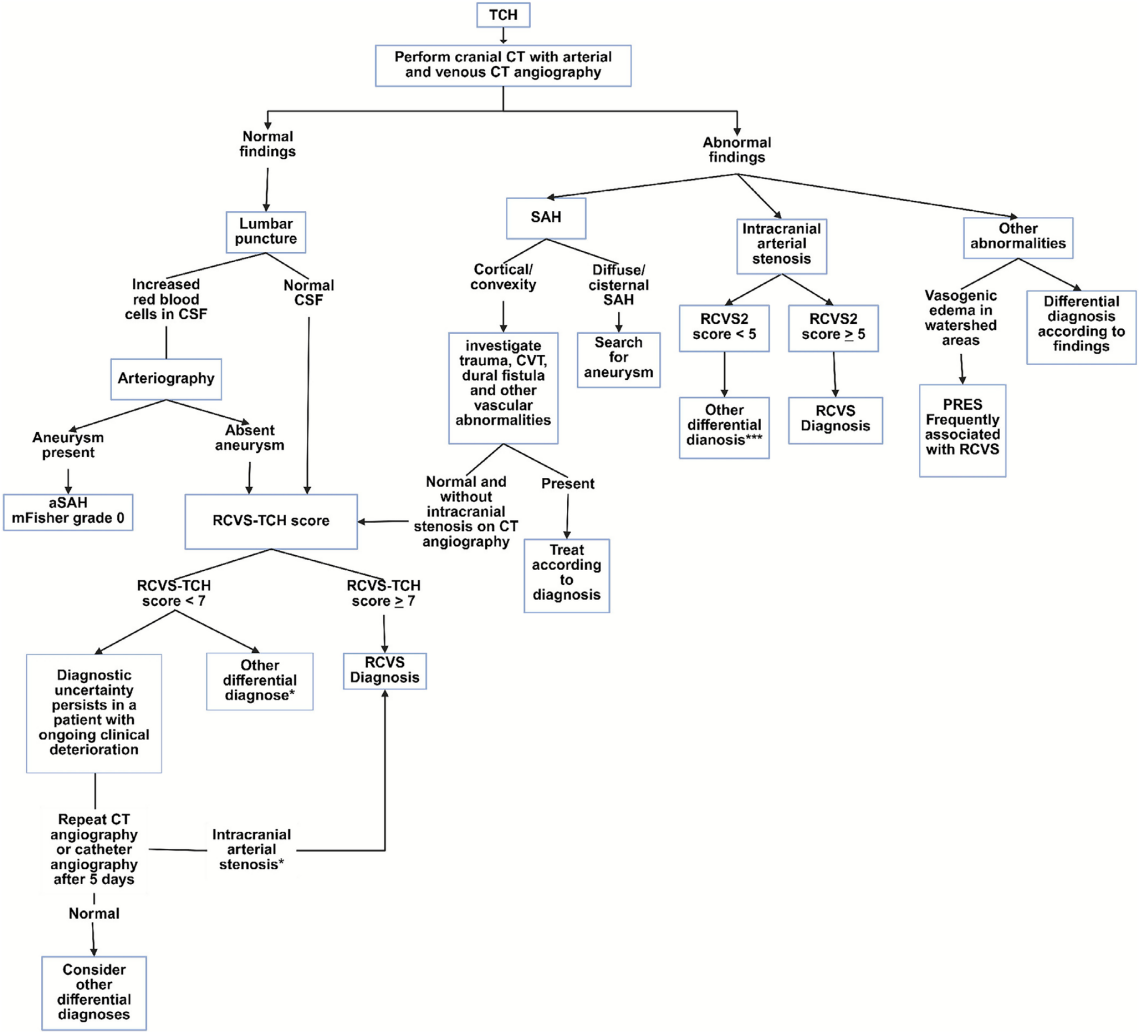
Approach to thunderclap headache and its differential diagnosis. [Color figure can be viewed at wileyonlinelibrary.com]

Other mimics of RCVS, such as CVT, intratumoral hemorrhages, and rupture of colloid cysts, are generally easier to identify after conducting a detailed clinical history and imaging studies. In cases of TCH with neuroimaging showing no structural alterations, performing a lumbar puncture is the next step. The analysis of CSF in these cases aids in identifying patients with modified Fisher grade 0 SAH (without evidence of bleeding on cranial CT) and meningitis. Furthermore, the addition of opening pressure evaluation assists in identifying low CSF pressure, which may be caused by a CSF fistula, a possible cause of TCH. For those cases with TCH where the analysis of CSF, neuroimaging, and vascular studies are completely normal, the diagnosis of RCVS cannot yet be ruled out; due to the centripetal pattern of arterial stenosis progression, patients in the acute phase of RCVS may exhibit stenoses only in the more distal vessels, which are not adequately assessed by vascular studies such as CTA. Therefore, repeating vascular imaging after 5–7 days may reveal the presence of arterial stenoses; however, given the typically benign and self‐limited course of the disease, this approach is rarely necessary. In patients with completely normal initial workup and low RCVS‐TCH scores, primary headache disorders, such as primary cough headache, Valsalva‐induced headache, or primary orgasmic TCH, should be considered in the differential diagnosis (Figure 5).

## MANAGEMENT

Currently, there is no proven disease‐modifying treatment for RCVS. The management of these patients is based on the identification and withdrawal of precipitating factors, along with symptomatic clinical support.

During the initial assessment, the active investigation of potential precipitating factors is of paramount importance so that they can be removed as early as possible (Table 1).

Blood pressure control is also a critical aspect because elevated blood pressure levels often occur concomitantly with the clinical syndrome and, in some cases, may act as a trigger for RCVS. There is no clearly defined optimal blood pressure target. In clinical practice, patients with RCVS and markedly elevated blood pressure levels (systolic blood pressure above 180–200 mmHg) are managed as hypertensive emergencies. Therefore, it is reasonable to adopt a cautious reduction strategy, aiming for a 20%–30% decrease in mean arterial pressure within the first two h of admission, followed by a more gradual control over the subsequent 24 h. Hypotension should be avoided due to the increased risk of precipitating ischemic events.^1^, ^137^

Pharmacological interventions with calcium channel blockers, such as nimodipine and verapamil, have been used in clinical practice. Evidence that these agents alter the natural course is lacking, although one study suggested that initiating nimodipine within 7 days of onset may reduce vasoconstriction.^138^ Their use persists due to the potential benefit of reducing the duration and intensity of headache.^19^ Other vasodilatory agents, such as magnesium sulfate and serotonergic antagonists, including dantrolene and cyproheptadine, have been described in small case series but with inconsistent results.^139^, ^140^ Corticosteroids are contraindicated in RCVS because they have been associated with clinical worsening, new radiologic lesions, angiographic progression, and poorer outcomes.^29^, ^108^, ^141^

Intra‐arterial vasodilator measures have been used as a therapeutic option in rare cases of symptom progression or worsening brain lesions despite clinical management. In such cases, balloon angioplasty and intra‐arterial infusion of nicardipine, milrinone, papaverine, or even nimodipine have been attempted, with variable outcomes.^123^, ^142^, ^143^ These interventions, in addition to being potentially associated with reperfusion syndrome, lack clear evidence of benefit and, therefore, should not be routinely employed in clinical practice.

Based on expert opinion, the resumption of daily activities that may trigger new events, such as sexual and physical activity, may be considered at least 1–2 weeks after complete clinical resolution.^1^ Medications with vasoconstrictive potential and known associations with RCVS should be avoided. However, in some clinical contexts, such as psychiatric conditions, the continuation of these medications may be imperative. In such scenarios, we suggest a slow and gradual reintroduction. Although there is no definitive evidence guiding this approach, clinical experience suggests that the gradual reintroduction of antidepressants and other vasoconstrictive agents after RCVS resolution is not associated with recurrence of TCH or angiographic abnormalities.^1^

## CLINICAL OUTCOMES

The overall prognosis of RCVS is excellent, with headaches and vasoconstriction typically resolving within days to weeks.^29^, ^133^ Long‐term outcomes are primarily influenced by complications such as ischemic stroke or hemorrhage, yet approximately 95% of hospitalized patients are discharged with a modified Rankin Scale (mRS) score of 0–2.^19^, ^29^, ^108^ Additionally, a recent cohort reported cerebral complications in only 5% of patients (all hemorrhagic), whereas about 84% achieved mRS 0 at 3 months.^144^ Persistent focal neurological deficits at follow‐up range from 3% to 20% in large series but are generally mild to moderate. Mortality rates vary across studies (0%–5%), with most reporting around 2%, predominantly in postpartum cases.^46^, ^107^, ^108^

Regarding post‐RCVS symptoms, more than half of patients experience chronic headaches persisting beyond 3 months after vasoconstriction resolution, with about half of affected patients requiring pharmacological treatment for up to 2 years.^145^ The pain phenotype was heterogeneous and distinct from the initial TCH, typically mild to moderate in intensity, most commonly bifrontal (52%), followed by left‐sided throbbing (20%), right‐sided throbbing (16%), vertex (8%), and occipital (4%). Concomitant symptoms included visual disturbances in 24% of patients, sensory symptoms in 8%, and weakness in 4%. In addition, a prior history of headache was the only baseline factor associated with this outcome.^146^ The recurrence rate of RCVS across independent large series is approximately 5%, with migraine, exercise, and sexual activity identified as independent predictors of relapse. Notably, most recurrences follow a benign course.^147^, ^148^

## CONCLUSION AND FUTURE PERSPECTIVES

Despite advances in understanding RCVS, significant gaps remain regarding its pathophysiological mechanisms, including the precise roles of endothelial dysfunction, autonomic dysregulation, genetic polymorphisms, and miRNA in the development of reversible vasoconstriction. The influence of sex, particularly the higher prevalence in women, suggests a possible hormonal impact on susceptibility to RCVS. However, this relationship remains poorly understood, and critical questions, such as how to prevent postpartum complications, are still unresolved. Additionally, the impact of concurrent infections and structural vessel abnormalities as potential triggers can make early recognition of the syndrome challenging and the true relationship with the overlap conditions requires further investigation. The REVERCE, a multinational, transcontinental cohort with rigorously phenotyped cases and standardized data collection, will provide the largest individual‐level dataset on RCVS to date. This task force aims to enable robust comparisons across regions, ethnicities, sexes, and age groups; clarify risk factors and triggers; and estimate functional outcomes and recurrence risk with greater precision.^149^

Given the association between RCVS and depression, careful management of serotonergic medications is essential, including dose adjustments or discontinuation when appropriate, to minimize the risk of recurrence. Similarly, the withdrawal of vasoactive drugs and substances known to trigger vasoconstriction is a crucial aspect of treatment, but the optimal strategy for managing patients who require continued use of these medications remains unclear.

From a prognostic perspective, although RCVS is generally considered a self‐limiting condition, a subset of patients develop complications, highlighting the need for research focused on identifying reliable clinical and epidemiological predictors of poor outcomes. A wide range of persistent symptoms in the chronic phase, including chronic headaches, fatigue, and mood disturbances, suggests that long‐term dysfunction may persist even after vasoconstriction resolves. Moreover, preliminary findings describe subtle cognitive impairment, which, along with other persistent symptoms, may define a post‐RCVS cognitive syndrome. However, further studies are needed to characterize the pattern of cognitive impairment and identify potential clinical or radiological markers associated with it.

Thus, future research is essential to clarify these aspects, improve disease and complication diagnosis, and refine follow‐up strategies for better management and interventions.

## AUTHOR CONTRIBUTIONS

**Ícaro Araújo de Sousa:** Conceptualization; writing – original draft; writing – review and editing. **Abner da Silva Machado:** Resources; writing – original draft. **Arthur de Oliveira Veras:** Resources; writing – original draft. **Thiago Oscar Goulart:** Visualization; writing – original draft. **Sarah Galassi Tatsuta:** Visualization; writing – original draft. **Trajano Aguiar Pires Gonçalves:** Visualization; writing – original draft. **Eva Rocha:** Writing – review and editing. **Octávio Marques Pontes‐Neto:** Writing – review and editing.

## CONFLICT OF INTEREST STATEMENT

**Ícaro Araújo de Sousa, Abner da Silva Machado, Arthur de Oliveira Veras, Thiago Oscar Goulart, Sarah Galassi Tatsuta, Trajano Aguiar Pires Gonçalves, Eva Rocha**, and **Octávio Marques Pontes‐Neto** have no conflicts of interest to declare.
